# A metagenomic study of biliary microbiome change along the cholecystitis‐carcinoma sequence

**DOI:** 10.1002/ctm2.97

**Published:** 2020-06-11

**Authors:** Xiaoling Song, Xu'an Wang, Yunping Hu, Huaifeng Li, Tai Ren, Yongsheng Li, Liguo Liu, Lin Li, Xuechuan Li, Ziyi Wang, Wen Huang, Runfa Bao, Yijian Zhang, Maolan Li, Xuefeng Wang, Feng Liu, Jun Gu, Linhui Zheng, Wenguang Wu, Yingbin Liu

**Affiliations:** ^1^ Department of General Surgery and Laboratory of General Surgery, Xinhua Hospital Shanghai Jiao Tong University School of Medicine Shanghai China; ^2^ Shanghai Key Laboratory of Biliary Tract Disease Research, Xinhua Hospital Shanghai Jiao Tong University School of Medicine Shanghai China; ^3^ Shanghai Research Center of Biliary Tract Disease, Xinhua Hospital Shanghai Jiao Tong University School of Medicine Shanghai China; ^4^ Department of General Surgery and Laboratory of General Surgery Xinhua Hospital, Shanghai Jiao Tong University School of Medicine 1665 Kongjiang Road Shanghai 200092 China; ^5^ Department of Biliary‐Pancreatic Surgery Renji Hospital, School of Medicine, Shanghai Jiao Tong University 160 Pujian Road Shanghai 200127 China; ^6^ Emergency Unit, The First Affiliated Hospital Nanchang University Nanchang China

## Abstract

**Background:**

Gallbladder cancer (GBC) is the most common cancer type of the biliary tract, and an association has been found between chronic calculous cholecystitis (CCC) and an increased incidence of GBC mortality. An understanding of the relationship between CCC and its carcinogenesis may enable us to prevent and cure GBC. In this study, we attempted to explore changes in the microbiome profile that take place during the transition from chronic cholecystitis mucosa to malignant lesions.

**Results:**

Seven paired human GBC and CCC samples were provided by patients who had undergone laparoscopic cholecystectomy or radical cholecystectomy. Mucosal DNA extraction and metagenomic sequencing were performed to evaluate changes in the microbiota between the two groups. We found that GBC patients and CCC patients shared similar stable and permanent dominant species and showed apparent differences in their biliary microbial composition and gene function. *Peptostreptococcus stomatis* and *Enterococcus faecium* may potentially play a role in GBC progression. In addition, the metagenomic species profiles, co‐abundance and co‐exclusion correlations, and CAZyme prevalence showed significant differences between the CCC and GBC groups.

**Conclusion:**

Our data suggested that changes in the microbiota between CCC and GBC may help deepen our understanding of the complex spectrum of different microbiotas involved in the development of GBC. Although the cohort size was small, this study has presented the first evidence of the existence of an altered biliary microbiota in GBC, which is clearly different from that in CCC patients.

## Graphical abstract

1. In the development of GBC, species richness and evenness are significantly increased compared with CCC group. 2. Abundance of NAD‐dependent protein deacetylase, deoxyribonuclease V, branched‐chain amino acid transport system permease protein and chorismate mutase were enriched in the GBC group. Archaeal S‐adenosylmethionine was enriched in the CCC group. 3. GHs, GTs, PLs and GEs were significantly higher in the CCC group, while GT77 was enriched in the GBC group.

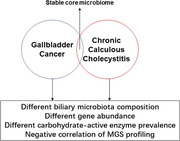

## 1 INTRODUCTION

Gallbladder cancer (GBC) is a highly invasive form of cancer and is the most common type of cancer of the biliary tract system.^1^, ^2^ Patients with GBC have an extremely bad prognosis, with 12‐month median and 5‐year survival rate of less than 5%. Surgical resection is the only option currently available treatment of GBC. However, most patients are diagnosed at advanced stages due to hidden clinical symptoms and the lack of specific biomarkers, and have already lost the possibility of undergoing radical resection. For patients who cannot undergo radical surgery or those with recurrent metastases, chemotherapy and radiotherapy produce unsatisfactory results.^3^, ^4^ The situation of GBC is severe, and the etiopathogenesis of GBC is not well understood. However, various factors, such as genetic susceptibility, infections, and lifestyle factors, have been thought to result in the occurrence of GBC. Recently, studies have found that the occurrence of epithelial malignant tumors, such as colorectal cancer,^5^ is associated with chronic inflammation. Interestingly, GBC has also been used as a model for a long time to understand the correlation between chronic inflammation and cancer.^6^ The presence of gallstones leads to periodic cell death and regeneration of the epithelial layer cells, which keeps the gallbladder in a constant inflammatory stimulated state.^7^ Since the 19th century, researchers have been concerned of the correlation between chronic cholecystitis (especially mixed gallstones) and GBC. Hsing et al reported that chronic gallstone disease contributes to a 21‐ to 57‐fold increase in the risk of developing GBC.^8^ Shrikhande et al^9^ also found that gallstones were associated with a high incidence of GBC. Therefore, understanding the relationship between chronic calculous cholecystitis (CCC) and its carcinogenesis may allow for the prevention and treatment of GBC.

Persistent bacterial infections may be responsible for chronic inflammation‐induced carcinogenesis. Mucosal surfaces exposed to the external environment are colonized by a vast number of microbes, and dysregulation of mucosal barrier function by microbiota and its consequences have been found to be associated with colorectal carcinoma progression.^10^ Moreover, distinct changes in mucosal microbial communities have been observed across the different colorectal carcinogenesis stages, and various members of the gut microbiota may be harbored by colorectal lesions.^11^, ^12^ Similar to the situation in the intestine, the biliary mucosa contains chemical, mechanical, and immunological barriers, which ensure immunological tolerance against commensals.^13^ Unsurprisingly, members of the phyla *Proteobacteria*, *Firmicutes*, and *Bacteroidetes* mainly populate the gallbladder ecosystem, and researchers have demonstrated the presence of intact bacteria within the biliary mucosa through microscopy.^14^ Moreover, chronic colonization of *S. Typhi* may be a primary predisposing factor for the onset of GBC.^15^ Specific host‐associated community assemblages are determined based on host cell composition and activity, molecular components of the mucus layer, and epithelial integrity.^16^ Thus, we raised the following question: how do microbiome profiles change during the transition from chronic cholecystitis mucosae to malignant lesions?

To address this question, we performed metagenomic sequencing of the mucosal microbiome of the CCC and GBC groups. In our study, we first identified human biliary mucosal microbiome signatures and then compared microbial community structure and function between the microbiota of the CCC and GBC groups. The results of our study may help deepen our understanding of the complex landscape of different microbiotas involved in the development of GBC.

## 2 MATERIAL AND METHODS

### 2.1 Sample collection

The Ethics Committee of Xinhua Hospital affiliated with Shanghai Jiao Tong University School of Medicine approved this study (No. XHEC‐D‐2019‐049). Approved guidelines were followed while performing the experiments. All patients provided informed consent for participation. The GBC specimens were provided by seven patients on whom radical cholecystectomy had been performed (without prior chemotherapy or radiotherapy), whereas the CCC specimens were provided by seven patients on whom laparoscopic cholecystectomy had been performed at the Department of General Surgery of the Xinhua Hospital Affiliated with Shanghai Jiao Tong University School of Medicine, China. The diagnosis of GBC and CCC was confirmed through hematoxylin and eosin (H&E) staining (Figure S1). The mucosal biopsies were collected, and the greatest dimension was at least 0.5 cm. Liquid nitrogen was used to snap‐freeze the biopsies immediately after cholecystectomy and the specimens were stored at –80°C.

### 2.2 DNA extraction

Bead‐beating was used to disrupt the mucosal biopsy samples, which were digested using an enzymatic cocktail of lysozyme and mutanolysin (Sigma). Then, a QIAamp DNA Mini Kit (Qiagen, Hilden, Germany) was used to extract and purify DNA from the samples by following the manufacturer's instructions. A NanoDrop spectrophotometer (Thermo Scientific, USA) was used to measure microbiomial DNA concentration. Thereafter, 0.5 μg/mL ethidium bromide‐added 1% agarose gel was used to detect the integrity and size of the extracted microbiomial DNA.

### 2.3 Metagenomic sequencing

The Illumina HiSeq × 10 platform (Illumina, Inc., USA) was used to sequence all samples. A paired‐end library was constructed using 500 bp as the size of the insert for each sample. A DNA LabChip 1000 Kit and an Agilent Bioanalyzer (Agilent Technologies, UK) were used for the evaluation of the quality of the libraries of each sample. Screening of the Illumina raw reads was conducted as follows: (a) removal of reads containing three ambiguous N bases; (b) trimming of reads containing low‐quality (*Q* < 20) bases; and (c) deletion of reads containing <60% high‐quality bases (Phred score ≥ 20). Then, a SOAPaligner (version 2.21) was used for the alignment of the clean reads to National Center for Biotechnology Information GenBank bacterial genomes.

### 2.4 Microbial relative abundance profiling and de novo assembly

The NCBI database was used for the alignment of the clean reads for the detection of known bacteria, fungi, viruses, and archaea. The aligned reads were classified by genus and species to determine classification and abundance. The taxonomy profile was constructed at different levels. Reads were assembled using SOAPdenovo (Version 1.05). For each sample, the reads were assembled using a series of k‐mers (51,55,59,63). At ambiguous Ns, the scaffolds assembled were split and contigs with more than 500 bp were selected for further analysis. Genes were predicted using MetaGeneMark software (http://exon.gatech.edu/GeneMark/metagenome/Prediction/). The predicted open reading frames were compared against the NCBI nonredundant sequence database using BLAST with default parameters.

### 2.5 Metagenomic species analysis

We clustered the genes into metagenomic species (MGS) based on differences in abundance between the samples. Then, a Spearman correlation coefficient (rho) of >.8 was used for the single‐strand clustering of differentially abundant genes (*P* < .05, Wilcoxon test). Clusters with a rho of >.8 were merged to obtain MGSs used to calculate the average abundance of clusters with over 25 genes. Based on the classification and relative abundance spectrum of the corresponding gene, classification annotation and abundance spectrum of each MGS were generated. A threshold greater than 90% of the genes in each MGS and the highest hit rate for the same phylogenetic group (>95% identical and >90% overlapping queries) were used to classify the MGS into taxonomic classifications that ranged from strain to superkingdom level.

### 2.6 Gene functional annotation and functional profiling

The databases, Carbohydrate‐Active enzymes (CAZy) and Kyoto Encyclopedia of Genes and Genomes (KEGG), were applied for the comparison between the assembled protein sequences and the annotated gene functions. In situations where the protein sequences were similar (*E*‐value <1 × 10^–5^ and score ≥60) to another protein sequence in the database, the assembled proteins were deemed to function in line with the proteins in the database. The most effective BLAST hit was used in the analysis. Thus, the different levels of functions were used to create the gene clusters.

### 2.7 Statistical analyses

R software (version 3.4.2) was used to perform all statistical analyses. Nonparametric Wilcoxon test was used to determine the statistical significance of the genes, KEGG orthologies (KOs), enzymes, and different taxonomic (phylum, genus, and species) levels. The enrichment characteristics with a *P* value of <.1 after adjustment were determined, and then determination of the enrichment group was based on the higher rank value. Benjamini and Hochberg's methods were used to adjust the *P*‐values of the false discovery rate (FDR). Ade4 and vegan (version 2.5‐1) were used for nonmetric multidimensional scale (NMDS), principal coordinate analysis (PCoA), and other Multivariate community diversity analyses, whereas ggpubr and ggplot2 were used to visualize the results. Calculation of species richness and the Shannon diversity index were conducted using the same software packages. The similarity index used was the Bray‐Curtis distance matrix. Heat map was used to conduct hierarchical clustering and Pearson correlation was used to establish the distance matrix. The most likely explanation for differences between the CCC and GBC groups (organisms, KOs, or (Ortholog GroOG (Ortholog Group) up) [OGs]) was determined through linear discriminant analysis (LDA) effect size (LEfSe) analysis. Differential features with a LDA score of <2.0 were noted.

## 3 RESULTS

### 3.1 Alteration of biliary microbiota composition in CCC and GBC patients

In order to detect the composition and function of biliary microbiota involved in the development of GBC, we performed metagenomic shotgun sequencing on mucosal biopsy samples collected from CCC and GBC patients (Tables 1 and 2). There are no significances in age, sex, gallstone number, and CCC duration between the CCC and GBC groups (Table 3, *P* > .05). High‐quality sequencing reads with an average of 10 GB/sample were generated. Species accumulation curves were captured to evaluate whether sequencing had sufficiently depicted the diversity of the biliary microbiome. As shown in Figure S2A, the curves of all samples were near saturation, which revealed that the sequencing depth and number were adequate.

**TABLE 1:** Demographic and clinical features of CCC patients

Sample	Age (year)	Sex	Disease duration (month)	Number of gallstone	The gallbladder grows or shrinks, gallbladder wall thickening
CCC‐01	60	F	3	1	Yes
CCC‐02	66	M	7	>3	Yes
CCC‐03	60	F	12	>3	Yes
CCC‐04	52	F	2	>3	Yes
CCC‐05	78	M	60	>3	Yes
CCC‐06	59	M	12	>3	Yes
CCC‐07	31	M	3	>3	Yes

**TABLE 2:** Demographic and clinical features of GBC patients

Sample	Age (year)	Sex	Whether with CCC	Number of gallstone	CCC duration (month)	TNM (AJCC 8th)	Differentiation degree	Neural invasion	Primary or metastatic	Tumor types
GBC‐01	71	M	No	0	0	III	1	No	Primary	Adenocarcinoma
GBC‐02	66	F	Yes	>3	24	IV	2	No	Primary	Adenocarcinoma
GBC‐03	40	M	Yes	>3	24	III	1	Yes	Primary	Squamous carcinoma
GBC‐04	78	M	No	0	0	IV	2	Yes	Primary	Adenocarcinoma
GBC‐05	66	F	No	0	0	III	1	No	Primary	Adenocarcinoma
GBC‐06	55	F	Yes	1	12	III	1	No	Primary	Adenocarcinoma
GBC‐07	46	F	Yes	>3	12	IV	2	No	Primary	Adenocarcinoma

Abbreviations: AJCC, American Joint Committee on Cancer; TNM, tumor node metastasis

**TABLE 3:** Comparison of CCC and GBC group

Parameter	Category	GBC (n)	CCC (n)	*P*‐value
Age	≥60	4	4	.766
	<60	3	3	
Sex	F	3	4	.626
	M	4	3	
Number of gallstone	0 ≥1	3 4	0 7	.055
CCC duration (month)	≥12 <12	4 3	3 4	.669

**FIGURE 1:**
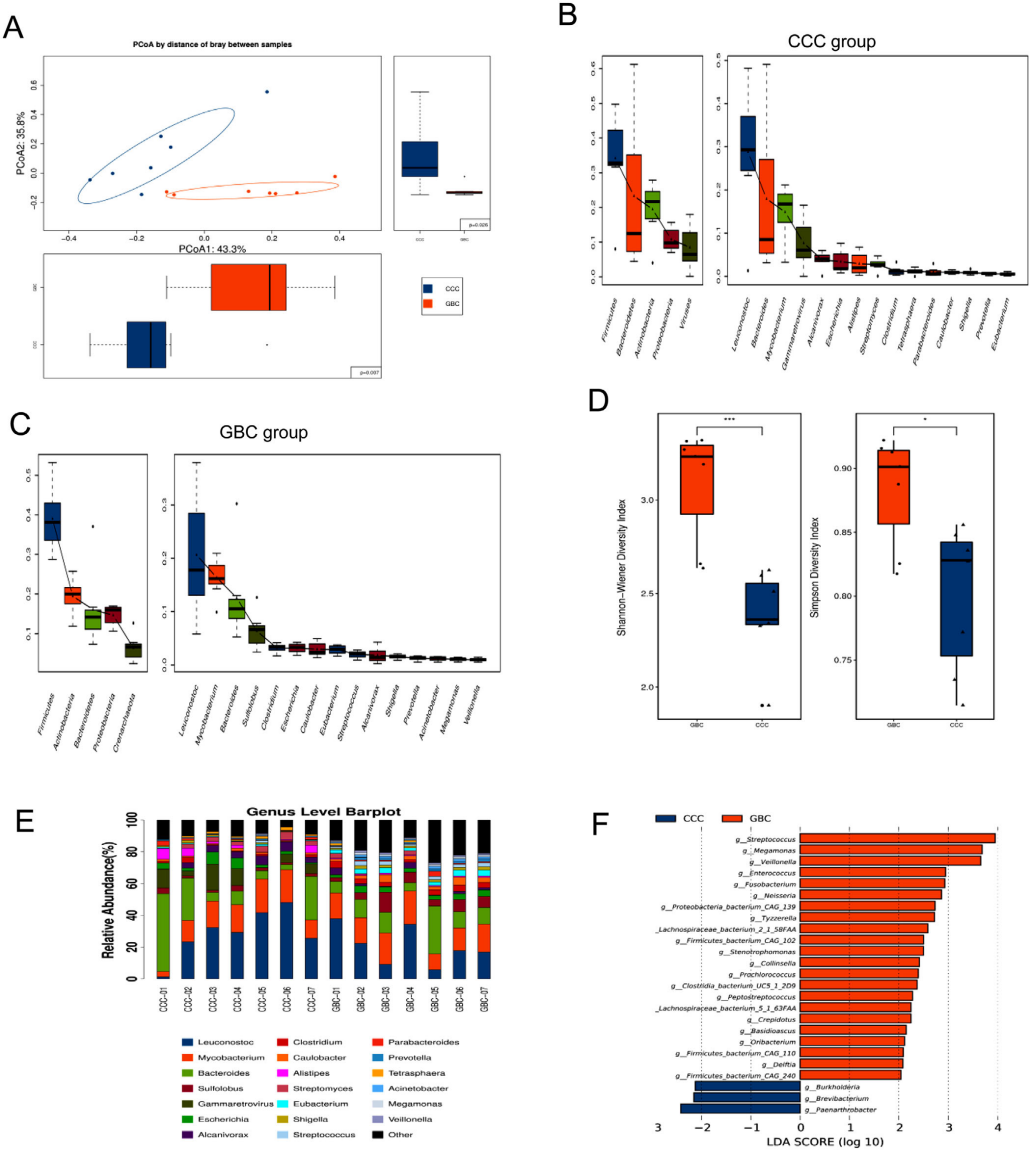
Alteration of biliary microbial composition in chronic calculous cholecystitis (CCC) and gallbladder cancer (GBC) patients. A, Principal coordinate analysis (PCoA) plot with Bray‐Curtis distances of CCC and GBC patients. B and C, Core microbiota generated from the genus abundance of CCC and GBC groups. D, The alpha diversity of the species richness and the evenness of the two groups as determined by the Simpson and Shannon‐Weiner indices. E, The diversity of biliary microbiota in two groups. The vertical axis represents genus abundance. F, Histogram of the linear discriminant analysis (LDA) scores computed for genera differentially abundant of CCC and GBC groups. The LDA scores (log10) > 2 are listed

We first investigated differences between the CCC and GBC samples using species data obtained through PCoA, analysis of similarities (Anosim), and NMDS analysis. As shown in Figures 1A, S2B, and S2C, significant differences were found in the structure of the mucosal microbiota between the CCC and GBC groups (*P* < .05). The “core microbiome” was used to identify and describe key microorganisms that were stable and permanent in each community.^17^ In our study, *Firmicutes*, *Bacteroidetes*, *Actinobacteria*, and *Proteobacteria* were found to be stable in both groups (Figures 1B and 1C), which is consistent with the results of a previous study,^14^ suggesting that severe biliary microbiome dysbiosis was not present in the study group at baseline. In order to further explore features of the biliary microbial community between the CCC and GBC groups, we measured the alpha diversity of species richness and evenness of the two groups using the Simpson and Shannon‐Weiner indices, respectively. As shown in Table S1 and Figure 1D, the diversity of the biliary microbiota was significantly lower in the CCC group, compared with the GBC group (Kruskal‐Wallis test, *P* < .05). To intuitively observe the species composition in each sample, histograms of genus and species abundance were created and are shown in Figures 1E and S2D. Moreover, LEfSe analysis was performed to examine differences in community composition between the groups, and features with an LDA score cutoff of 2.0 were considered as different. The results show the top 25 species with the highest level of significant variation (Figures 1F and S2E). At the genus level (Table S2), *Basidioascus*, *Peptostreptococcus*, *Crepidotus*, and *Fusobacterium* and at the species level (Table S3) *Peptostreptococcus stomatis*, *Fusobacterium mortiferum*, *Acinetobacter junii*, and *Enterococcus faecium* were found to be positively correlated and significantly contributed to the GBC group cluster. Hence, our analysis revealed that GBC patients and CCC patients shared stable and permanent dominant species, whereas they displayed apparent differences in biliary microbial composition, which may account for the relationship between the two groups.

### 3.2 Microbial gene function differed between the CCC and GBC groups

**FIGURE 2:**
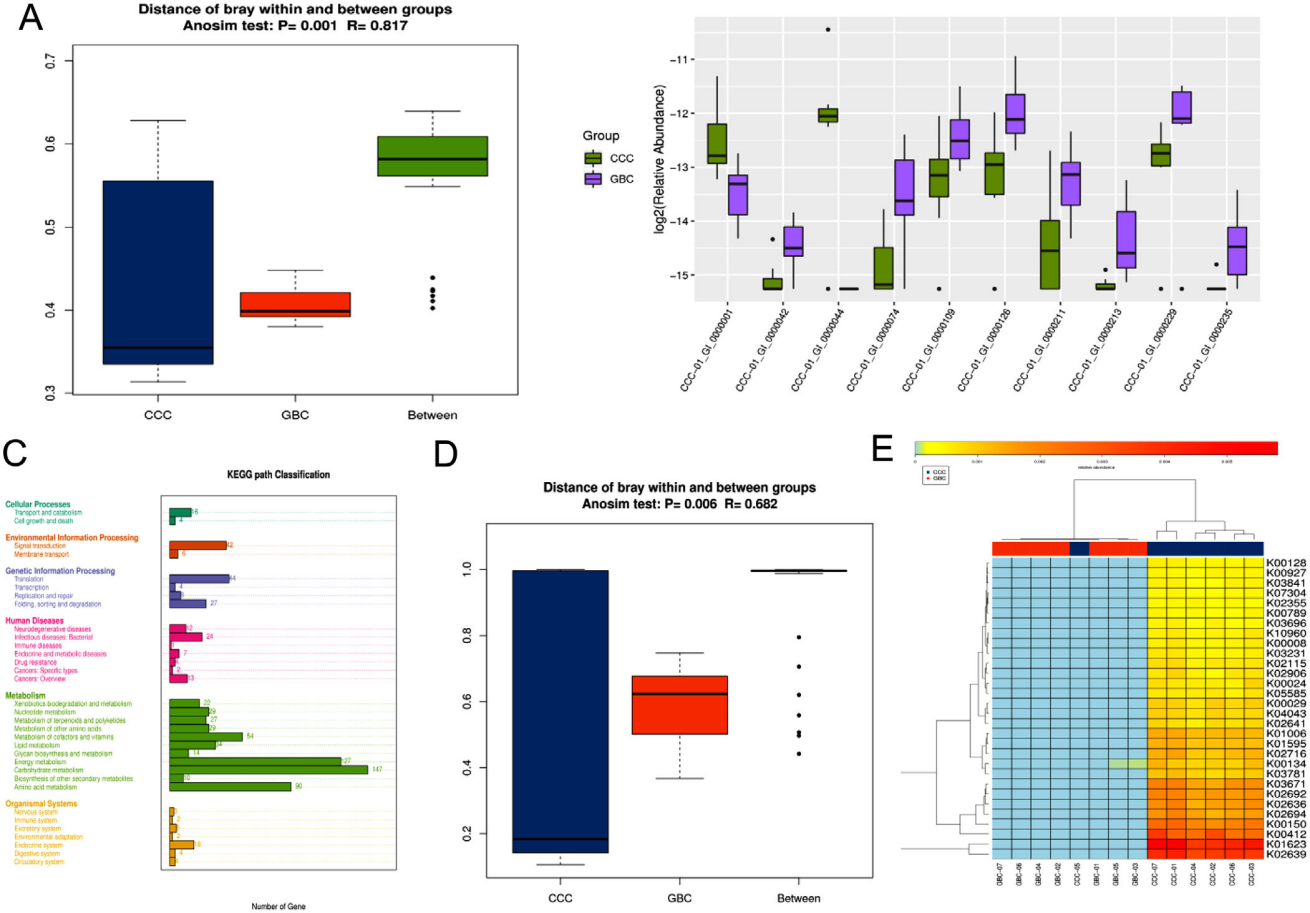
Microbial gene function differed between the chronic calculous cholecystitis (CCC) and gallbladder cancer (GBC) groups. A, Anosim analysis of microbial gene function differed between the CCC and GBC groups. The abscissa represents the category of the group, and the ordinate represents the distance between the samples. B, Differences in gene abundance between the biliary microbiota of the two groups. C, KEGG pathway classification of the two groups. D, Anosim test of significant difference relative abundance of KEGG orthology (KO). E, Majority difference relative abundance of KO of the two groups

To explore signatures of the biliary microbiota in the CCC and GBC samples, we assembled filtered data using metaSPAdes based on a De‐Brujin graph and predicted functional genes using the MetaHIT database. The genes identified were compiled into a nonredundant catalogue of 19 927 genes, which allowed for genes with greater than 500 bp of the reads in each sample to be mapped. The Anosim test performed using Bray‐Curtis distance suggested an obvious difference in the composition of the biliary microbiota between the CCC and GBC groups (Figure 2A, *R*‐value = .817, *P*‐value = .001). Gene abundance was also compared between the biliary microbiota of the two groups. As shown in Figure 2B, the abundance of a NAD‐dependent protein deacetylase, deoxyribonuclease V, branched‐chain amino acid transport system permease protein, and chorismate mutase was enriched in the GBC group, whereas Archaeal S‐adenosylmethionine and some hypothetical proteins were enriched in the CCC group.

We annotated the 19 927 genes using the KEGG functional database^18^ to investigate the microbial functions that influenced each of the two groups. As shown in Figure 2C, most of the genes were involved in metabolism activity, especially energy and carbohydrate metabolism. Next, we illustrated whether the microbiota contributed to different functions by comparing the mean relative abundance for each KO between the groups. The Anosim, principal component analysis (PCA), and PCoA test results revealed significant differences in the relative abundance of the KOs (Figures 2D, S3A, and S3B), which was consistent with the results of a previous study.^11^ Furthermore, we observed that most KOs in the GBC group were present at low proportions. Although CCC‐05 showed results similar to GBC group, we suspected that it was more prone to malignant changes due to severe inflammation (Figure 2E). To reveal differences in community composition between the two groups, LEfSe analysis was performed. The results showed that oligosaccharide 4‐alpha‐D‐glucosyltransferase and Fe‐S cluster assembly protein SufB were more abundant in the GBC group (Figure S3C).

### 3.3 MGS profiling of the CCC and GBC groups

**FIGURE 3:**
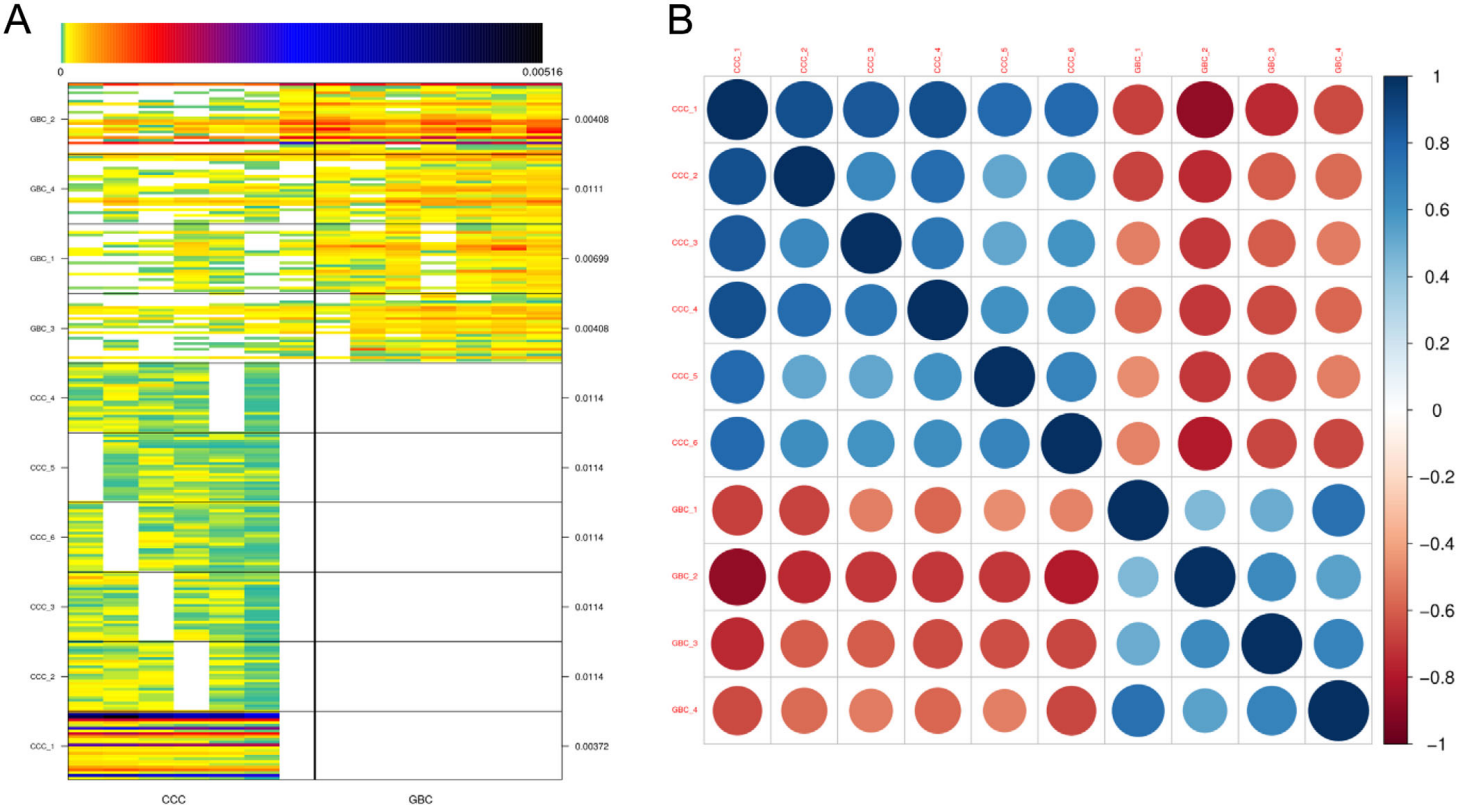
Metagenomic species (MGS) profiling of the chronic calculous cholecystitis (CCC) and gallbladder cancer (GBC) groups. A, Heat map of MGS profiling. The abscissa represents the sample, and the vertical axis represents the 25 genes with the highest abundance in each MGSs. B, Relationship networks of MGS profiling. The point size and color depth represent the correlation between MGS, the blue represents the positive correlation, and the red represents the negative correlation

The microbial components of the CCC and GBC groups were investigated using MGS profiling. A total of 2543 microbial genes that were obviously different between the two groups were identified (Table S4). Subsequent relative abundance profiling identified 10 MGSs, of which four were enriched in the GBC group, whereas six were enriched in the CCC group (Figure 3A). As shown in Figure 3B, the MGS profiles of CCC and GBC showed significant negative correlation, which indicated a difference between the two groups.

### 3.4 Detection of the interactions of differentially abundant microbes between CCC and GBC groups

**FIGURE 4:**
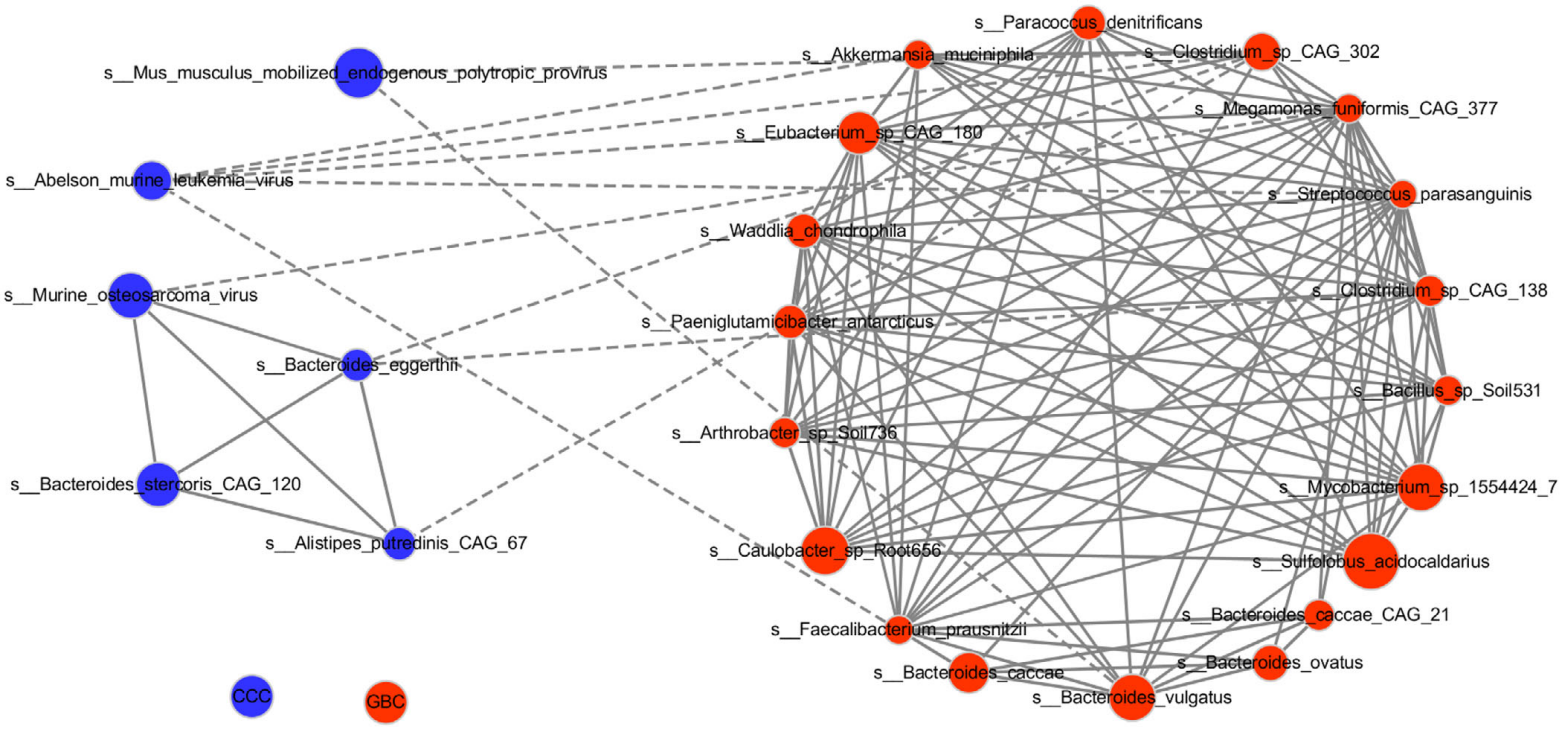
Detection of interactions of differentially abundant microbes between chronic calculous cholecystitis (CCC) and gallbladder cancer (GBC) groups. SparCC network plot of co‐abundance and co‐exclusion correlations between the CCC and GBC groups

In order to evaluate potential interactions by the differentially abundant microbes between the two groups, a SparCC network plot of co‐abundance and co‐exclusion correlations was created. As shown in Figure 4, the two groups formed their own mutualistic networks, which were distinctly different. Moreover, the significant correlation logarithm and correlation intensity among the GBC bacteria were higher than that of CCC bacteria. All the above results suggested the presence of complex interaction networks among these microbes, although more research is needed to explore these interactions.

### 3.5 Influence of the CCC and GBC microbiomes on carbohydrate‐active enzyme prevalence

**FIGURE 5:**
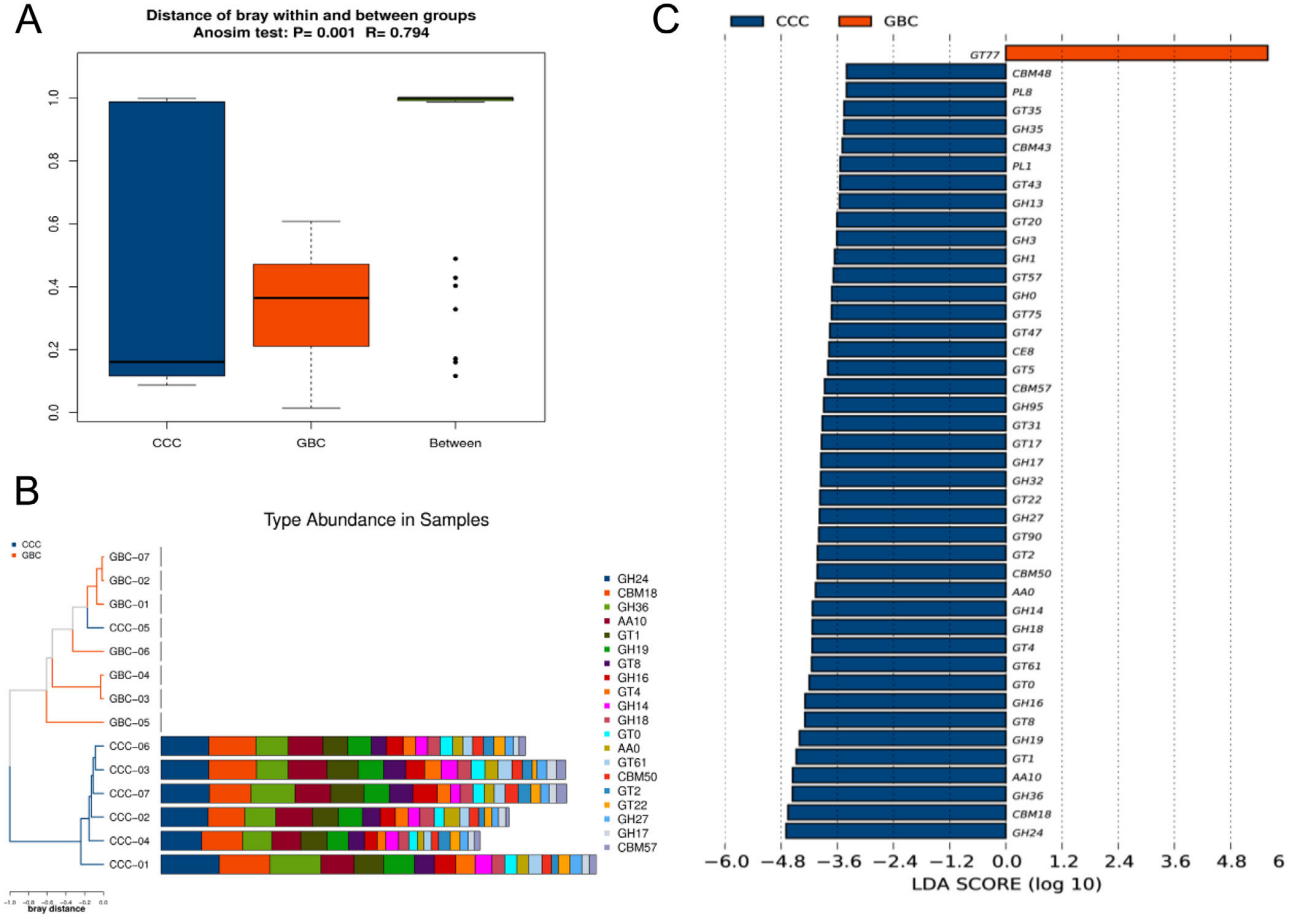
Influence of the chronic calculous cholecystitis (CCC) and gallbladder cancer (GBC) microbiomes on the carbohydrate‐active enzymes (CAZymes) prevalence. A, Anosim analysis of the CCC and GBC microbiomes on CAZ prevalence. B, Histogram of the relative abundance of different enzyme types in each sample on the CAZymes prevalence. C, Histogram of the LDA scores computed for different enzyme types in each sample on the CAZymes prevalence

As shown in previous results (Figure 3), the level of carbohydrate metabolism was found to be high in the biliary microbiota. We screened for carbohydrate‐active enzymes (CAZymes) in assembled contigs to explore their potential for complex carbohydrate degradation in the CCC and GBC biliary metagenomes. First, Anosim, PCoA, and PCA tests were performed to demonstrate the carbohydrase composition in the two groups. As shown in Figures 5A, S4A, and S4B, an obvious difference was found between the CCC and GBC groups. Moreover, the CCC group showed higher diversity of CAZymes than the GBC group, which is consistent with the species and KO abundance results (Figure 5B). Furthermore, LEfSe analysis results demonstrated that GHs, GTs, PLs, and GEs were significantly enriched in the CCC group, whereas GT77 was enriched in the GBC group. All these results revealed a decreased capacity for complex carbohydrate metabolism in the CCC group.

## 4 DISCUSSION

The microbiome has attracted significant attention due to its influence on human diseases, including cancer, in recent years. Cardiovascular diseases, mental diseases, digestive diseases, and many other types of diseases have been found to be associated with microbiome dysbiosis.^19^, ^20^, ^21^ Recently, its role in cancer has become increasingly apparent, with the microbiota involved in about  20% of human malignancies.^22^ A growing number of studies have demonstrated the role of the microbiota in the carcinogenesis of various cancers, such as colorectal, liver, breast, and pancreatic cancers.^23^, ^24^, ^25^ However, it is unknown whether biliary microbiota dysbiosis can induce GBC carcinogenesis.

A study was performed on a cohort of 396 720 South Korean men and women, which revealed that gallstones were associated with an increased risk of GBC mortality and was independent potential confounder for the disease.^26^ Genetic and environmental factors include diet‐ and metabolism‐induced gallstone formation. The persistent existence of gallstones may cause repeated damage to the gallbladder mucosa, which leads to nonintact epithelial layer cells and chronic cholecystitis. Some pathogenic bacteria tend to colonize the gallbladder mucosa through nonintact mucous membranes, inducing chronic inflammation infection. Colonization of the gallbladder by *Helicobacter* spp. and *Salmonella enterica* serovar Typhi has been classically associated with gallstones and chronic infections.^27^, ^28^ Moreover, the link between chronic inflammation and cancer is clear, and approximately 18% of all human cancers have been linked to precancerous inflammation.^29^ Therefore, clarification of the features of microbiome colonization in CCC and GBC mucosa may help us better understand the onset of GBC carcinogenesis. Research on the microbiome in diseases has mainly focused on fecal microbiota because of the convenience of obtaining noninvasive biological samples and technical limitations.^30^ The difficulty in obtaining solid biopsies or liquid fluids and the lack of adequate methods to evaluate the molecular structure of microbial ecosystems with a low bacterial load have hampered the identification of other human microbial niches. Until now, the microbiota of the human gallbladder, especially in GBC, has rarely been studied. Fortunately, Natalia et al characterized bile samples from the gallbladder and found that the gallbladder harbors a microbial ecosystem.^31^ Nevertheless, the characterization of the human gallbladder mucosa microbiota has not been performed. In this study, we deciphered microbial signatures implicated in gallbladder tumorigenesis by performing metagenomic shotgun sequencing of mucosal biopsy samples collected from CCC and GBC patients, which is the first study of its kind.

Colonization by pioneer microorganisms and the diversity of microorganisms in the gastrointestinal tract play an important role in establishing a symbiotic system of host‐microbial interactions.^32^ Previous studies have focused on bacterial populations of patients with cholecystolithiasis or cholecystitis, but spectrum changes in GBC flora have been only poorly described. In this study, we first attempted to explore changes in colonizing bacteria during the progression of CCC to GBC. We found that the core microbiota was similar in both groups, whereas during the development of GBC, species richness and evenness decreased significantly. Apparent differences were also observed in biliary microbial composition. *Peptostreptococcus stomatis*, *Fusobacterium mortiferum*, and *Enterococcus faecium* were found to be positively correlated and significantly contributed to the GBC group cluster, which might account for the relationship between the two groups. Among them, *Peptostreptococcus stomatis* and *Enterococcus faecium* drew our attention. *Peptostreptococcus stomatis* was enriched and was shown to play a potentially important role in colorectal and gastric cancer progression.^33^, ^34^ *Enterococcus faecium* may exert a mechanistic impact on anti‐PD‐1 efficacy in metastatic melanoma patients.^35^ This prompted us to consider that *Peptostreptococcus stomatis* and *Enterococcus faecium* may potentially play important roles in GBC progression. Their association and impact need to be evaluated using further studies. The number of samples included in the two cohorts in our study was limited, due to difficulties in obtaining a sufficient number of GBC samples for metagenomic sequencing, and we will attempt to broaden the number of samples in the two cohorts to verify our results in the future. In addition, we also attempted to depict differences in colonizing bacteria between GBC tissues and adjacent tissues. However, due to the small size of the gallbladder itself and cancer tissue encroachment on the entire gallbladder due to its late stage, it was difficult to obtain a sufficient quantity of adjacent tissues.

The mechanisms by which the microbiota contributes to carcinogenesis by affecting the risk of the host can be divided into three categories: change in the balance of host cell proliferation and death, by affecting immune system function, or by affecting the metabolic functions in the host.^36^ Our analysis revealed that certain functional changes were associated with bacterial enrichment. Based on the results of our study, we found that the abundance of NAD‐dependent protein deacetylase, deoxyribonuclease V, branched‐chain amino acid transport system permease protein, and chorismate mutase was elevated in the GBC group, whereas the abundance of archaeal S‐adenosylmethionine and certain hypothetical proteins was elevated in the CCC group. Bacterial changes in CCC and GBC may lead to changes in certain functional gene families and pathways that contribute to gallbladder carcinogenesis. The metabolic functions of the microbiota associated with GBC remain largely unclear. In our study, we also explored the potential of complex carbohydrate degradation in CCC and GBC biliary metagenomes. GHs, GTs, PLs, and GEs were significantly upregulated in the CCC group, whereas GT77 was significantly upregulated in the GBC group, which revealed a decreased capacity for complex carbohydrate metabolism in the CCC group. However, the mechanism through which changes in the levels of functional genes or complex carbohydrates influence the development of GBC from CCC is still unknown. In addition, our study did not thoroughly explore whether biliary microbiota contributes to carcinogenesis by affecting host immune system function. Therefore, further in‐depth and effective research studies are needed to verify and further explore the results of this study.

## 5 CONCLUSION

Overall, we discovered distinct compositions of the biliary microbiome in CCC and GBC patients and compared the microbial community structure and function of the microbiota of each microbiome in relation to disease pathogenesis. Although the cohort is limited in number, our study demonstrated a novel approach in understanding the mechanism of GBC tumorigenesis by clarifying changes in mucosal community composition during the development of GBC. However, our research is still insufficient. Future genomic analyses need to be conducted to explore interactions between immune cell populations, host cell epigenomes, and microbiomes, which are critical to determine the multifaceted role of the biliary microbiota in human health and disease.

## ETHICS APPROVAL AND CONSENT TO PARTICIPATE

This study was approved by the Ethics Committee of Xinhua Hospital affiliated with the Shanghai Jiao Tong University School of Medicine (No. XHEC‐D‐2019‐049). All experiments were carried out in accordance with the approved guidelines. Informed consent was obtained from the patients.

## AVAILABILITY OF DATA AND MATERIALS

The datasets used and/or analyzed during the current study are available from the corresponding author on reasonable request.

## CONFLICT OF INTEREST

The authors declare no conflict of interest.

## AUTHOR CONTRIBUTIONS

Xiaoling Song, Xu'an Wang, Wenguang Wu, and Yingbin Liu conceived and designed the study. Xiaoling Song, Yunping Hu, Huaifeng Li, Tai Ren, Yongsheng Li, Liguo Liu, and Lin Li collected and processed the biological samples. Xiaoling Song, Xuechuan Li, and Ziyi Wang developed the DNA extraction protocol. Xiaoling Song, Wen Huang, Runfa Bao, and Qin Zhu analyzed the metagenomic data. Yijian Zhang, Maolan Li, Xuefeng Wang, and Xiaoling Song prepared the figures and tables. Xiaoling Song, Yunping Hu, Feng Liu, and Linhui Zheng wrote the manuscript. Xiaoling Song and Jun Gu edited the manuscript. All authors read and approved the final manuscript.

## Supporting information

Supporting Information.

Supporting Information.

Supporting Information.

Supporting Information.

Supporting Information.
