# Supplementary material for: A metagenomic study of biliary microbiome change along the cholecystitis‐carcinoma sequence
Source: Clin Transl Med. 2020 Jun 11;10(2):e97. doi: 10.1002/ctm2.97 (PMC7403721; doi:10.1002/ctm2.97)
Supplement: Supplementary file 5 — Supporting Information. [file CTM2-10-e97-s005.docx]

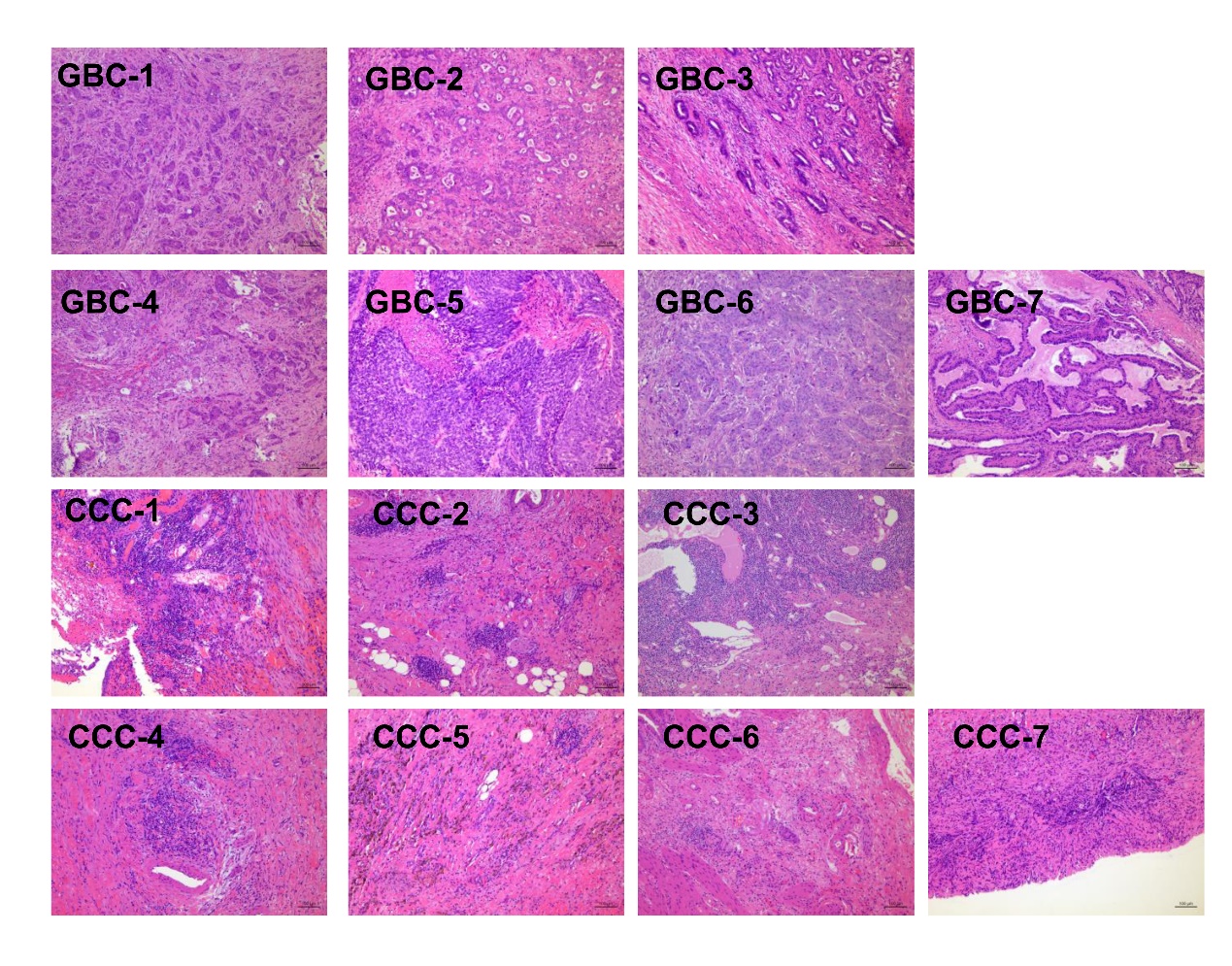
Figure S1 HE staining of chronic calculous cholecystitis （CCC）and gallbladder cancer (GBC) patients.


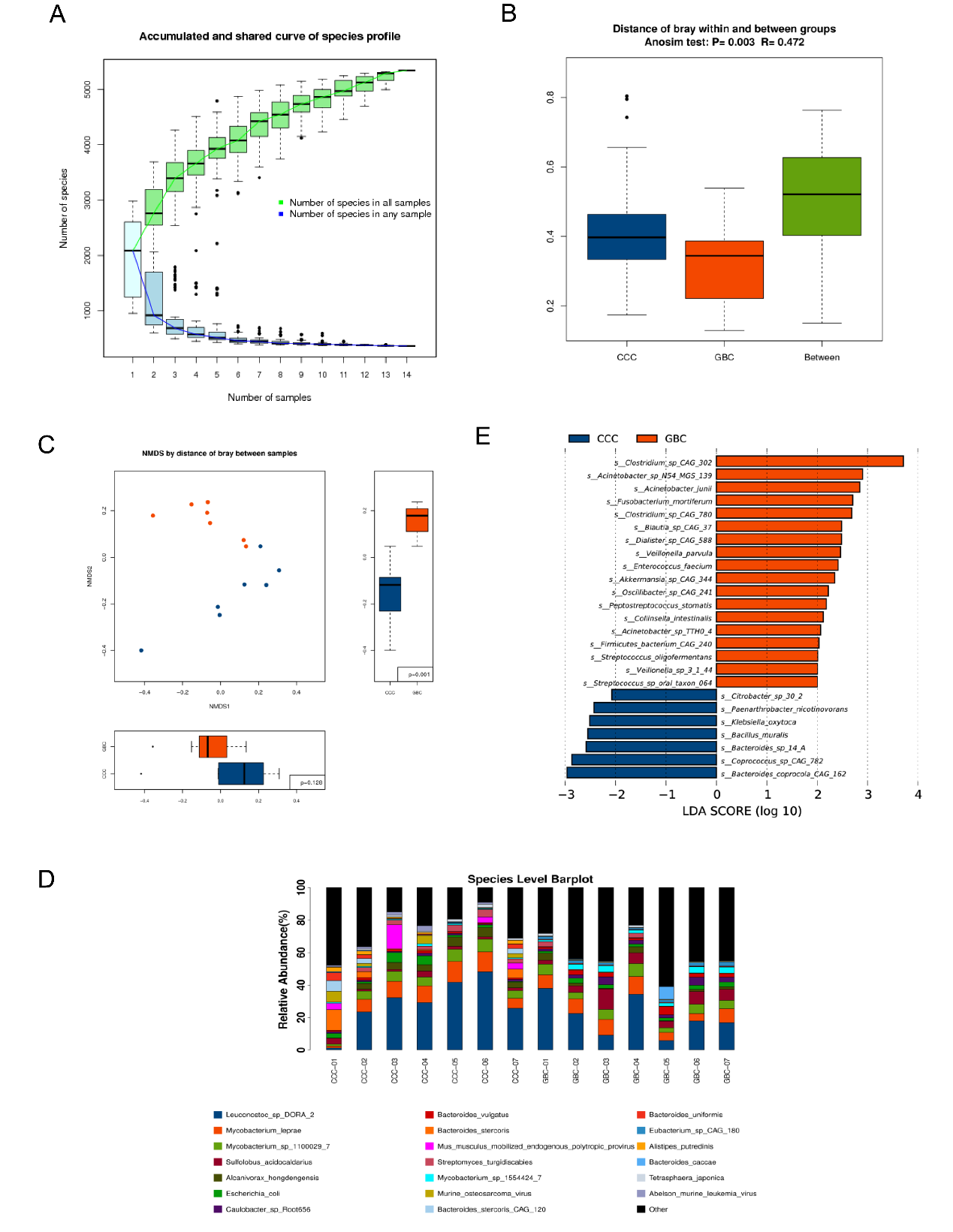
Figure S2. A) Species accumulation curves of the samples. The abscissa represents the sample number, and the ordinate represents the number of species after sampling. B) Principal component analysis (PCA) and nonmetric multidimensional scaling (NMDS) of chronic calculous cholecystitis （CCC）and gallbladder cancer (GBC) patients. C) The diversity of the biliary microbiota in two groups. The vertical axis represents species abundance. D) Histogram of the linear discriminant analysis (LDA) scores computed for differentially abundant species in the CCC and GBC groups. The LDA scores (log10) > 2 are listed.


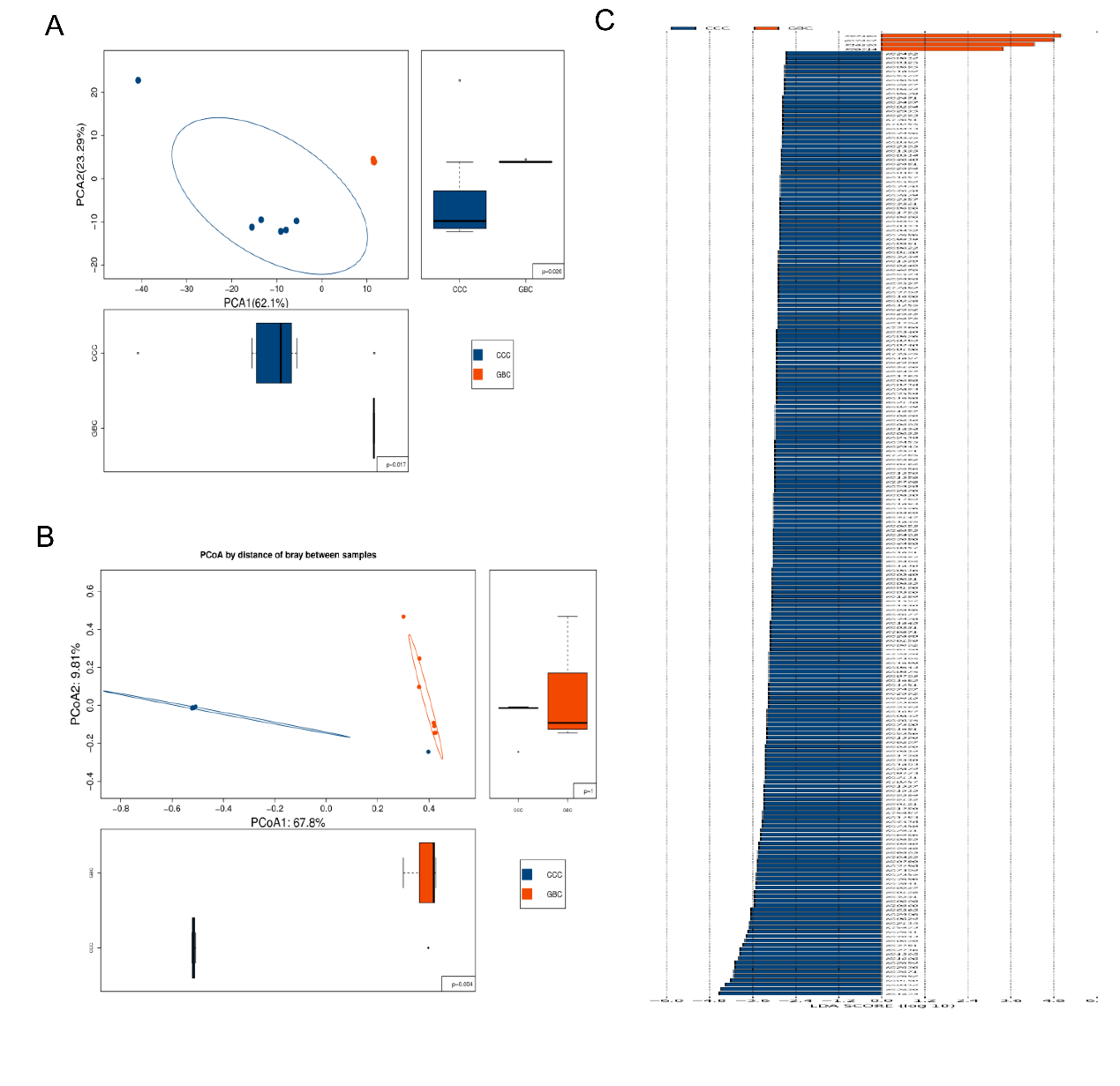
Figure S3. A-B) Principal coordinate analysis (PCoA) and principal component analysis (PCA)analysis of microbial gene functions differing between the chronic calculous cholecystitis(CCC）and gallbladder cancer (GBC) groups. C) Histogram of the linear discriminant analysis (LDA) scores computed for microbial gene functions differing between the CCC and GBC groups.


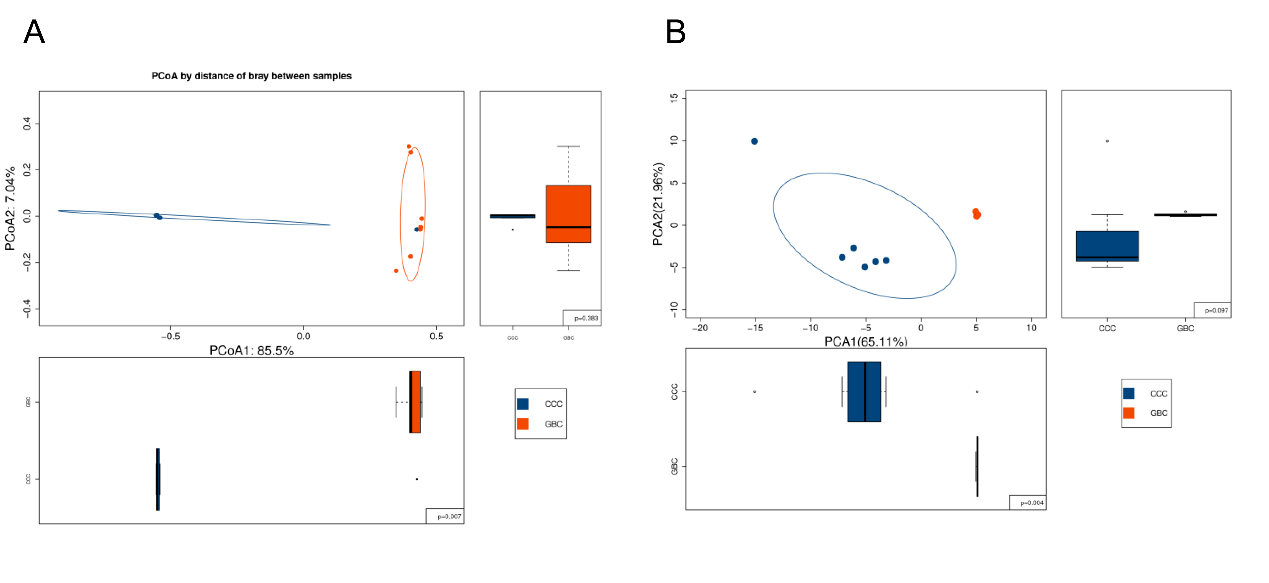
Figure S4. A-B) Principal coordinate analysis (PCoA) and principal component analysis (PCA) analysis of chronic calculous cholecystitis(CCC）and gallbladder cancer (GBC) microbiome on the ~~CAZyme~~ carbohydrate-active enzymes (CAZymes) prevalence.
